# Performance Estimation of a Medium-Resolution Earth Observation Sensor Using Nanosatellite Replica

**DOI:** 10.3390/s24103160

**Published:** 2024-05-16

**Authors:** Carlos Colodro-Conde

**Affiliations:** Instituto de Astrofísica de Canarias, c/Vía Láctea s/n, E-38205 La Laguna, Spain; ccolodro@iac.es

**Keywords:** performance estimation, earth observation, nanosatellite, multispectral, short-wave infrared (SWIR), InGaAs, signal-to-noise ratio (SNR), dynamic range

## Abstract

In many areas of engineering, the design of a new system usually involves estimating performance-related parameters from early stages of the project to determine whether a given solution will be compliant with the defined requirements. This aspect is particularly relevant during the design of satellite payloads, where the target environment is not easily accessible in most cases. In the context of Earth observation sensors, this problem has been typically solved with the help of a set of complex pseudo-empirical models and/or expensive laboratory equipment. This paper describes a more practical approach: the illumination conditions measured by an in-orbit payload are recreated on ground with the help of a replica of the same payload so the performance of another Earth observation sensor in development can be evaluated. The proposed method is specially relevant in the context of small satellites, as the possibility of having extra units devoted to these tasks becomes greater as costs are reduced. The results obtained using this method in an actual space mission are presented in this paper, giving valuable information that will help in further stages of the project.

## 1. Introduction

Space exploration has undergone profound changes in recent years. While in the past, only large governmental agencies could access space, advances in the miniaturization of technology and reduced launch costs, among other factors, have opened access to space to new agents, both private and public. In addition, the new paradigm has enabled more agile innovations in all aspects of a space mission, not only related to technology, but also to the applied methods and processes [1,2].

In the field of Earth Observation (EO), the latest trends point to further improvements in spatial, spectral and temporal resolution [3,4]. Achieving this objective from orbit with acceptable image quality is a remarkable challenge, due to the complex interplay of the many parameters that are involved [5,6]. As a result, designers of EO payloads should consider image quality metrics from early stages of the project, leveraging both theoretical models and empirical testing to ensure the feasibility and effectiveness of their designs.

Historically, space missions have emphasized rigorous testing regimes, adhering to a “model philosophy” that involves the iterative testing of development units before finalizing the flight units. The idea is to produce and test several units of the products under development, before the final flight units are produced. Early testing becomes even more important at the current stage of space exploration, where missions tend to rely on innovative technologies in order to push the boundaries of their respective fields. In these cases, the early validation of design concepts is critical to mitigate the perception of risk that is typically associated with technologies with lower maturity levels [7].

This study was performed in the context of a new EO payload under development by the IACTEC-Space group, part of the Instituto de Astrofísica de Canarias (La Laguna, Spain). The payload consists on a multispectral imager covering from visible to short-wave infrarred (SWIR) wavelenghts, aiming to reach resolutions down to 5 meters per pixel with minimum volume, mass and power consumption. The design is focused on exploiting the broad bandwidth of extended-range InGaAs sensors, which are still a novelty in the context of EO missions, with the first radiation-resistant products having appeared quite recently [8]. Following the guidelines of the European Space Agency (ESA) onh the adoption of new technologies [9], it was decided within the project that testing should start at early as possible with the construction of a functional prototype. This prototype would be illuminated in a way that is representative of the one expected in orbit, so that imaging quality parameters can be obtained directly from the acquired images.

One way to determine the intensity and shape of the spectrum that should illuminate the prototype is to use theoretical models that consider aspects such as the Sun’s emission spectrum, the transmittance of the atmosphere for a given set of conditions, the optical properties of the target, the geometry of the observation, etc. [10]. Despite the undeniable value of such models, their large number of input parameters can add some level of uncertainty, as sometimes, it is difficult to determine an adequate set of parameters for each specific case. The method presented in this paper prioritizes the use of actual in-orbit measurements over the values estimated by models, taking advantage of a situation that can happen in the context of a mission: a fully representative replica of an in-orbit EO payload is also available on ground. This was just the case of the mission concerning the present paper.

The main idea of the proposed method is to prepare an illumination system that makes the ground replica produce the same response as the in-orbit payload does while pointing to a given target under certain illumination conditions. As will be explained later, the use of real Sun illumination as a reference makes the results more reliable than those that could be obtained in the laboratory with artificial light sources and a similar level of effort. Specialized systems for characterizing Earth Observation sensors are certainly available [11,12], but their high complexity might place them out of the reach of small satellite missions, which tend to be tight on budget. On the other hand, commercial off-the-shelf solutions like solar simulators [13], which are typically used for measuring the performance of solar cells in the photovoltaic industry, would not be able to provide the level of spectral match required for most EO payloads, due to the narrow filter bandwidths that they typically use [14].

The rest of this paper is divided into the following sections. Section 2 (“Materials”) presents the components that will be used throughout the paper. Section 3 (“Methodology”) defines the proposed method and applies it to the payload under study. Section 4 (“Results”) describes the final test set-up and lists the results that were obtained from it. Section 5 (“Discussion”) analyzes the obtained results and their implications on subsequent iterations of the design. Finally, Section 6 (“Conclusions”) summarizes the key ideas presented throughout the paper while also identifying the areas that might be subject to improvement.

## 2. Materials

This section describes the materials involved in the present studies. They were used to illustrate the application of the proposed method with an actual example. This example addresses the characterization of a new Earth Observation payload (VINIS, “Visible Near-Infrared and SWIR”), using another payload with flight heritage (DRAGO-2, “Demonstrator for Remote Analysis of Ground Observations”) as a reference. These two payloads, developed by the IACTEC-Space group as part of its EO programme, will be described in Section 2.1 and Section 2.2, respectively. On the other hand, Section 2.3 will present an additional component that will be used to reflect light in a controlled way.

### 2.1. Device under Test

**Figure 1:**
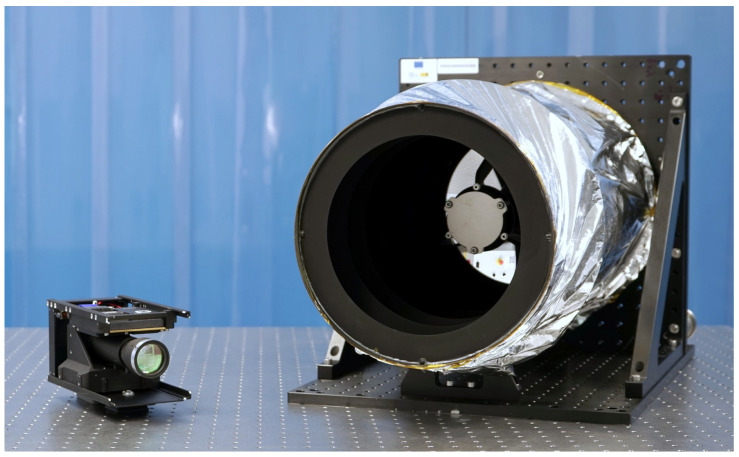
Space cameras used in the present studies. From left to right: DRAGO-2 and functional prototype of VINIS (VINIS-PROTO).

The example device under test (DUT) was VINIS-PROTO, a functional prototype of VINIS (“Visible Near-Infrared and SWIR”) [15,16]. VINIS is a multispectral payload designed to take 5 m/pixel images from LEO (Low Earth Orbit) in the visible, NIR (Near-Infrared) and SWIR (Short-Wave Infrared) wavelengths, with intended applications ranging from firefighting support to the water stress monitoring of crops. A picture of VINIS-PROTO, along with the rest of the cameras developed at IACTEC-Space, can be found in Figure 1. The main specifications of VINIS can be found in Table 1, which also includes the specifications of DRAGO-2.

One of the key design decisions that were made for VINIS is that it would use a single 2-D InGaAs detector to cover the whole spectral range defined for this payload. The type of detector that VINIS uses is typically referred to as an “extended response” sensor. This sensor has a special manufacturing process (InP substrate removal) that makes it differ from standard InGaAs devices, which are only sensitive to SWIR wavelengths (between λ≈0.9 μm and λ≈1.7 μm) [17,18,19,20,21].

The use of InGaAs detectors for covering such a wide spectral range is the main reason that motivated the creation of a functional prototype. Indeed, former medium-resolution payloads have used dedicated sensors for visible + NIR bands (e.g., Si-based CCDs) and dedicated sensors for SWIR (e.g., deep-cooled HgCdTe detectors). The functional prototype was built to measure performance parameters, and their compliance with the defined requirements might be at risk due to this design decision.

In contrast to traditional multi-sensor payloads, the use of a single detector that does not require deep cooling helped in reducing the volume, mass and power consumption of VINIS, as it eliminates the need for bulky radiators and/or cryogenic systems to lower the temperature to operational levels [22,23]. In addition, having a single sensor covering the whole spectral range enables simplifications in the associated control and readout electronics, which would otherwise have to handle the specific needs of different sensor technologies by means of dedicated circuitry.

Table 2 shows the list of bands included in VINIS-PROTO. The filters that implement these bands were assembled together to form a single piece and then placed at a close distance to the InGaAs surface (≈1 mm) with the help of a custom filter holder. The selected filter pieces had not been specifically designed for VINIS, but were built using leftover material that was available at that time from past projects. Given this situation, the parameters shown in Table 2 do not match perfectly with the values originally intended for VINIS; in particular, the filter for the blue (“B”) band did not reject SWIR wavelengths properly, causing the sensor to detect those undesired wavelenghts as well. Still, the rest of the filters served just fine for the purposes of the present study, and the obtained results proved to be useful for making decisions at further stages of the project, as explained later in Section 5 (“Discussion”).

### 2.2. Reference Payload

This study used DRAGO-2 [24,25,26] as the reference payload. For some context, DRAGO (“Demonstrator for Remote Analysis of Ground Observations”) is the name of the series of space cameras developed at IACTEC-Space to observe the Earth in SWIR wavelenghts from LEO. DRAGO-2 is the second version of the cameras, designed to provide finer GSD values. A picture of DRAGO-2 can be found in Figure 1, while its main specifications are listed in Table 1.

At the time of writing the present document, two units of DRAGO-2 had been launched to space, both with successful results [25,26]. The reference images used in the present study were taken from the first unit: the one onboard the ION SCV-007 satellite, launched on January 2023. Unlike the later unit, which had two observing bands, the selected unit had a single band centered at the upper part of the sensitivity range of the InGaAs sensor, around 1623 nm. Among other uses, this band is known to be useful in measuring the moisture content of soil and vegetation [27]. As it is so close to the definition of the B11 band of Sentinel-2 MSI (MultiSpectral Instrument), it will be certainly possible to compare images from the two payloads. As a reference, Table 3 shows a comparison of the two observing bands.

### 2.3. Calibrated Reflector

As explained later in Section 3.1.1, it was assumed that ground targets follow the properties of Lambertian reflectors. In the proposed test set-up, a reflection over this type of surface was simulated by means of a Spectralon target using a Labsphere (Labsphere, Inc., North Sutton, NH, USA) model SRT-20-050. These targets have a nominal reflectance of 20% and a size of 12.7 cm × 12.7 cm. According to the calibration curves given by the manufacturer, the actual reflectance values of this specific target are the ones listed in Table 4. All the details about how this target was used in the final test set-up are given in Section 3.4.

## 3. Methodology

The objective of the proposed method is to prepare a test set-up that illuminates the device under test (DUT) with light that is representative of the desired in-orbit scenario. An accurate reproduction of the expected spectrum shape will be achieved by reflecting sunlight over a calibrated target. Then, the target will be tilted until the reference payload on ground gives the same readings as its in-orbit counterpart. At this point, it will be possible to replace the reference payload with the DUT, so an estimation of the in-orbit performance can be obtained by performing direct measurements. In other words, the list of steps that comprise the proposed method are as follows:

1. *Definition of the Reference Scenario*: the key parameters for the case under study shall be identified and quantified so that they can be considered for the selection of the in-orbit reference image(s). At a minimum, it is recommended to consider the reflectance properties of the observed target and the observation geometry.
2. *Selection of the Reference Image(s)*: the dataset of in-orbit imagery of the reference payload shall be inspected to find the closest match to the parameters established in the previous step. If the reflectance properties of the observed targets are not known, images from another calibrated payload should be obtained as well.
3. *Analysis of Reference Image(s)*: the image of the reference payload shall be analyzed to extract the value that will be used as a reference for the payload replica on ground. A valid option can be the raw data provided by the image sensor, expressed in DNs (digital numbers), just as provided by its ADC (Analogue-to-Digital Converter).
4. *Preparation of the Test Environment*: a calibrated reflector shall be used to reflect sunlight in such a way that the payload replica on ground provides the reference value established in the previous step. Then, the DUT shall be put in the place of the payload replica.
5. *Data Acquisition*: with the DUT already receiving an input spectrum that is representative of the defined reference scenario, a proper set of measurements shall be taken to allow the derivation of the desired performance parameters.

Just as mentioned before, the proposed method will be applied to an actual example, where the DUT is VINIS-PROTO and the reference payload is DRAGO-2 onboard ION SCV-007. Additional supporting imagery will be obtained from the Sentinel-2 mission [28]. Each step of the proposed method will be elaborated on in a dedicated subsection hereafter. To provide a general overview, the general steps are particularized as follows:

1. *Definition of the Reference Scenario*: first, the key parameters of the case under study will be identified by analyzing their influence on the incoming light. Then, specific values will be assigned to those parameters, using the island of Tenerife as a reference.
2. *Selection of the Reference Image(s)*: a proper target and its corresponding image will be selected from DRAGO-2 in-orbit imagery. Calibrated Sentinel-2 images from that day will be downloaded as well, so as to know the reflectance of the selected target.
3. *Analysis of Reference Image(s)*: the image taken by DRAGO-2 will be analyzed to obtain the in-orbit reference value. In addition, the raw DRAGO-2 image will be compared to the corresponding Sentinel-2 image in order to establish a relationship between raw values given by DRAGO-2 and actual target reflectance values.
4. *Preparation of the Test Environment*: a test set-up will be prepared outdoors to make a replica of DRAGO-2 give the same values that DRAGO-2 gave in orbit when it acquired the reference image. In this way, the spectrum received on ground at that point of space and time will be similar to the one that DRAGO-2 received from orbit.
5. *Data Acquisition*: with the input spectrum already tuned as a result of the previous step, sets of images will be acquired for each observation band of VINIS-PROTO to enable the extraction of performance parameters like the signal-to-noise ratio (SNR) or the dynamic range.

### 3.1. Definition of the Reference Scenario

These studies will use the island of Tenerife (Canary Islands) as a reference to evaluate the performance of VINIS. This island is one of the main regions of interest for the VINIS mission, as well as the location of the IACTEC-Space headquarters. The choice of this location determines the range of some parameters that affect the amount of light that reaches the payload, as will be explained in the following paragraphs.

There is a considerable number of parameters that affect the amount of light received by an Earth Observation payload, including hard-to-predict factors such as the abundance of specific molecules (H_2_O, O_3_, CO_2_, etc.) throughout the atmosphere. Considering every possible parameter would detract from the focus of the present study, which aimed to follow a practical approach that prioritizes actual measurements over estimations obtained using complex models like MODTRAN [29].

In order to find an adequate balance between practicality and accuracy, it was decided that the present study would focus on the parameters that would affect the amount of received light to a greater extent. A sensitivity analysis was performed over the parameters that have most commonly been considered in the literature [6,30,31], giving the results summarized in Table 5. From these results, it was decided that the present study would assess the reflectance properties of the observed target and the observation geometry, which have a higher influence on the received radiance than the rest of parameters.

As a summary of the discussions included in the following subsections, the reflectance of the observed targets will be assumed to be around 0.10 in the visible bands and around 0.25 in the NIR and SWIR bands. These represent typical reflectance values of the island of Tenerife. On the other hand, the Solar Zenith Angle (SZA) will be assumed to be around 54°, which is a representative value for the observation geometry in Tenerife during winter. Considering this large SZA as a reference will allow checking whether the required SNR can be reached even under demanding illumination conditions.

#### 3.1.1. Reflectance Properties

The observed objects will be assumed to be Lambertian surfaces, reflecting light like ideal diffusers. This is a common assumption in the field of Earth Observation, including the visible and SWIR regions [10,34,35,36], although it is true that the reflectance properties of an object can be better described using more complex models [6,36,37,38].

The properties of Lambertian surfaces state that the amount of reflected light at a given wavelength just depends on the reflectance of the object itself (ρ) and the angle between the normal of the surface and the incident light rays (θ), as described in the following equation:

(1) $$I_{r}=\rho I_{i}\cos\theta$$

where $I_{i}$ is the incident radiant intensity, and $I_{r}$ is the reflected radiant intensity.

The amount of light reflected by the target will thus depend strongly on the illumination angle and the reflectance of the object itself. If there was nothing affecting the propagation of the reflected light, as in vacuum conditions, the apparent brightness would be the same regardless of the observation angle, thanks to the perfect diffusing properties that have been assumed. Note that the case of Earth Observation needs to consider the effect of the atmosphere on the reflected light rays, as explained later in Section 3.1.2.

Regarding the expected reflectance of the objects observed from orbit, the scientific objectives of VINIS established that this payload should be able to obtain SNRs higher than 60 for all its observation bands while pointing to areas with “typical” reflectance values. In order to determine such values, Sentinel-2 L2A (Level-2A) images of Tenerife [39] were downloaded and analyzed, giving average SR (Surface Reflectance) values of around 10% for visible bands and 25% for NIR and SWIR bands. These will be the reference values that were used later when estimating SNRs, as discussed in Section 4.2.

#### 3.1.2. Observation Geometry

In looking at Equation (1), it is clear that the illumination angle (θ) will play an important role in the amount of light received by the satellite. However, the equation does not consider the fact that light rays can travel through the atmosphere for different distances depending on their angle with respect to the zenith. The key parameters here are the SZA (Solar Zenith Angle) and the OZA (Observation Zenith Angle).

**Figure 2:**
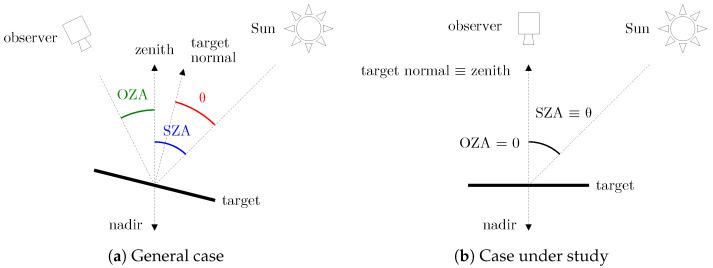
One-dimensional representation of the angles that define the observation geometry, namely, the SZA (Solar Zenith Angle), the OZA (Observation Zenith Angle) and the illumination angle (θ). (**a**) General case, with no specific assumptions about the illumination geometry. (**b**) Case under study, where the observation direction is aligned to the nadir, and the target normal is aligned to the zenith.

For simplicity, the reference scenario will assume nadir observation (OZA $\approx 0^{{}^{\circ}}$), which is the expected attitude mode of an Earth Observation satellite. On the other hand, it will be assumed that the normal of the observed surface is aligned to the zenith (i.e., 0° slope terrain), making the illumination angle (θ) equal to the SZA. Figure 2 shows a representation of the angles involved both in the general case and in the case under study.

The assumptions made for the case under study leave the SZA as the angle that will affect the observation to a higher extent, for two reasons. Firstly, because it will determine the amount of reflected light given by Equation (1), with the SZA being equal to θ. Secondly, because it will determine the path of light rays in the downward direction, thus affecting the total amount of losses that the light experiences as it passes through the atmosphere.

There are several models available in the literature for estimating atmospheric losses, featuring various levels of complexity. The most simple models are based on the concept of “air mass” (AM). The air mass can be defined as the ratio between the length of the path that follows the actual light ray and the length of the path in the case of an object at sea level illuminated from the zenith. For angles below 60°, its value can be simply estimated using a plane-parallel atmosphere model [40]:

(2) $$\mathrm{AM}=\frac{1}{cos(\mathrm{SZA})}$$

Regarding the atmospheric losses, the model in [32] provides the following estimation:

(3) $$I_{g}=I_{0}\cdot 0.7^{\left({\mathrm{AM}}^{0.678}\right)}$$

where $I_{g}$ is the irradiance at ground level (after passing through the atmosphere), and $I_{0}$ is the extraterrestrial irradiance (before entering the atmosphere).

To know the range of SZA values that may occur while observing Tenerife from orbit, the SZA values of each day throughout one year were calculated. The chosen time for this study was 12:00 UTC, which is close to the average passing times of the Sentinel-2 satellites over the island. This yielded a minimum SZA of 15.727° (summer) and a maximum SZA of 54.094° (winter). In combining Equations (1)–(3), the total attenuation factor between the best case (minimum SZA) and the worst case (maximum SZA) is around 1.9. This number gives an idea of how much the light that reaches an Earth Observation payload can vary just by changing the SZA. Changing other parameters like the altitude of the target over the sea level can make this factor reach values of 2.0 or more [33].

### 3.2. Selection of the Reference Image(s)

With the reference scenario already defined, the first step of the proposed method is to select a proper pair of reference images (one from DRAGO-2 and another one from Sentinel-2) of the same region of Earth, so that they can be later compared.

In order to ensure the best possible results, the images were chosen so that they included large uniform areas with approximately flat elevation profiles (i.e., with constant elevation). This simplifies the process of co-aligning images, as DRAGO-2 images would not need to be ortho-rectified with a DEM (Digital Elevation Model). In addition, the constant slope given by a flat surface would make the illumination conditions constant throughout the areas of interest, thus avoiding the need to further apply DEM-based corrections to remove the effect of rugged terrain. Finally, images were required to have cloud coverage close to 0%, not only to avoid the visual obstruction of the surface but also to minimize the effect of atmospheric phenomena that can affect the measurements, such as the presence of haze.

The requirements mentioned above could not be fulfilled for island of Tenerife, due to its rugged and heterogeneous terrain. In contrast, it was rather likely to fulfill these using images of desert areas. After going through desert images in the DRAGO-2 dataset, it was decided that the reference DRAGO-2 images would be the ones acquired on 28 February 2023 at 06:17:15 UTC over the Iranian desert. Besides satisfying all the aforementioned conditions, it happened that Sentinel-2A passed barely one hour later over the same region, at 07:19:21 UTC. This last feature was thought to be very convenient, as it would minimize the changes that may happen between the selected pair of reference images, due to either changes in the target itself (e.g., surface humidity) or in the illumination conditions (e.g., shadows cast by the Sun at different sky coordinates).

**Figure 3:**
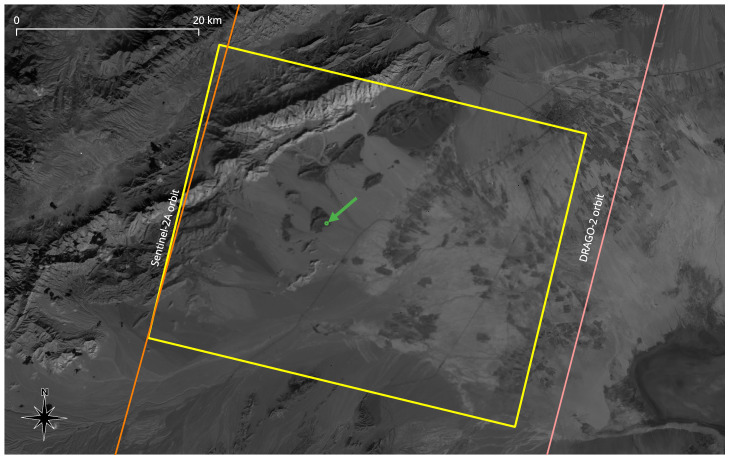
Reference images used for the present study, both around 1600 nm. The bottom raster layer corresponds to the Sentinel-2 MSI B11 band at the L1C processing level. The top raster layer, highlighted with a yellow border, corresponds to the DRAGO-2 image, with its histogram adjusted to match the bottom layer. Orbit footprints are shown as vector lines. The ROI is highlighted in green.

The selected images can be found in Figure 3, both around 1600 nm, as defined in Table 3. In this figure, the base Sentinel-2 layer comes from L1C (Level-1C) products [41], meaning that the value of each pixel is proportional to the TOA (Top-Of-Atmosphere) reflectance at the corresponding coordinate. The selected DRAGO-2 image was processed up to a similar processing level, with the exception that it was not properly ortho-rectified to consider the effect of the terrain profile on pixel placement. Given the relatively flat profile of the imaged region, this fact did not affect the results of the present study.

Besides showing the reference images themselves, Figure 3 represents the orbit footprints corresponding to these passes, calculated from the TLEs (Two-Line Elements) corresponding to that day. The same figure also defines the ROI (Region Of Interest) that will be carefully analyzed in the following subsections: a circular region with 300 m of diameter located at coordinates +36.054, +54.062 (that is, 36°03′14″ N, 54°03′43″ E).

### 3.3. Analysis of Reference Image(s)

The analysis of the reference images was performed with the QGIS software, version 3.28.15. QGIS [42] is a free and open source GIS (Geographic Information System) that covers all the needs of the present analyses.

#### 3.3.1. Observation Angle

During the pass over the ROI, the distance between orbit footprints was 45.5 km. Sentinel-2 was pointing to the nadir, according to its nominal scanning mode. DRAGO-2 was pointing with a certain off-nadir angle, as deduced using the central coordinates of the full-frame image shown in Figure 3. In addition to this, both satellites were seeing the ROI from a different observing angle.

The first question that arises from the facts mentioned in the paragraph above is whether the different angles will considerably affect the amount of light received by each satellite. To help clarify this point, Table 6 shows a list of relevant parameters. The first three parameters were obtained from the geometry given by the map in Figure 3 and the TLE-based orbit estimation. The atmospheric attenuation factor refers to additional losses with respect to a nadir observation, due to the fact that the light rays in the upward direction need to go through a thicker layer of atmosphere. This is just the number that was added to the last row of the table, obtained by applying Equations (2) and (3).

The obtained attenuation factors, both very close to unity, show that the satellite pointing has a negligible effect in these studies, so they will be ignored in further calculations.

#### 3.3.2. Effect of Terrain Profile

It was mentioned in Section 3.2 that flat targets (i.e., targets with their surface normals close to the zenith direction) would be preferred in order to simplify the analysis. The actual situation over the selected target is analyzed in this section, trying to determine up to which extent such simplifications can be applied in this case.

**Figure 4:**
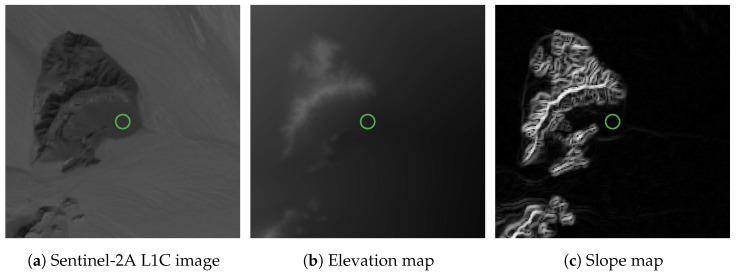
DEM-related maps for regions around the ROI (green circle). (**a**) Sentinel-2A L1C reference image (B11 band), with brighter pixels indicating more incoming light. (**b**) Elevation map (directly from DEM), with brighter pixels indicating higher altitudes. (**c**) Slope map (derived from DEM), with brighter pixels indicating steeper terrain.

First, Figure 4 shows various grayscale representations of features related to the DEM around the selected ROI, obtained from the Copernicus GLO-30 dataset [43]. A visual inspection of the maps in this figure reveals that inside the defined ROI, all of the parameters of interest (amount of received light, elevation profile and slope) appear to be approximately constant. In addition, the slope seems to have a low value, judging by the dark values in the slope map. The quantitative analysis results presented in Table 7 confirm that the ROI has a high degree of flatness, just as intended.

As a reference, Table 8 lists the general illumination parameters during both passes. The attenuation factor given in this table is only accurate for objects with a 0° slope, but already hint at a higher levels of reflected light during the Sentinel-2 pass. For this reason, the following section will base its calculations on Sentinel-2 L2A (Level-2A) products, where the effect of illumination angles have been corrected with the help of DEMs, in contrast to Sentinel-2 L1C (Level-1C) products [30].

#### 3.3.3. Target Reflectance

The reflectance of the target itself was assumed to be equal to the SR (Surface Reflectance) values given by the Sentinel-2A L2A images. Table 9 lists some statistics calculated for the ROI, while also comparing them to the values given by DRAGO-2. The standard deviation of the reflectance values confirms that the selected ROI was indeed a good choice, as it appears to reflect light uniformly. Note that the analyzed bands are those listed in Table 3, making the numbers obtained from both satellites comparable with each other.

#### 3.3.4. Cross-Calibration

The analysis of satellite imagery in the previous subsections not only provided confidence that the selected target had the desired properties for the present study but also presented some numbers that are the key for the cross-calibration process. In the context of the present study, the objective of the cross-calibration process was to obtain a simple formula that can convert the 14-bit DNs (digital numbers) given by DRAGO-2 into reflectance values.

Before diving into a radiometric calibration, it is important to note one property of the sensor on-board the DRAGO cameras, which will simplify the calculations. According to laboratory tests performed before launch, consisting of exposure time sweeps under constant illumination, the sensor is highly linear (linearity better than 99%) for practically its whole dynamic range. This means that as long as the pixels are not close to saturation, the DN values given by DRAGO will be proportional to the reflectance of the target, provided that they are corrected from DSNU (Dark-Signal Non-Uniformity) and PRNU (Photon-Response Non-Uniformity) effects. In the present study, DRAGO-2 DSNU was corrected using the method described in [44], while the PRNU was corrected based on flat-field images taken in the laboratory while pointing to an integrating sphere illuminated by a quartz halogen lamp. Bad pixels were not corrected, but they were nevertheless avoided in the ROI so that the obtained statistics were not altered.

Table 8 and Table 9 indicate that DRAGO-2 achieved a 4820.1 DN (DSNU- and PRNU-corrected) while pointing to a relatively-flat target with a 0.246 reflectance, with the Sun located 54.85° away from the zenith. The two latter numbers match, very closely, the values determined in Section 3.1, just as originally intended. For similar SZAs and similar atmospheric conditions, the corrected DN value obtained using DRAGO-2 can be predicted as follows:

(4) $$y_{t}=\rho_{t}\frac{y_{r}}{\rho_{r}}$$

where $y_{t}$ is the corrected DN value provided by DRAGO while pointing to the desired target, $\rho_{t}$ is the reflectance of the desired target around 1600 nm, $y_{r}$ is the corrected DN value given by DRAGO while pointing to the reference target ($y_{r}=4820.1$ DN), and $\rho_{t}$ is the reflectance of the reference target around 1600 nm ($\rho_{r}=0.246$). The coefficient between these reference values is the so-called “calibration factor”. In DRAGO-2, this value is equal to 19,593.902 DN. It corresponds to the value that the sensor would present while pointing to a target with 100% reflectivity, without considering saturation effects.

### 3.4. Preparation of the Test Environment

**Figure 5:**
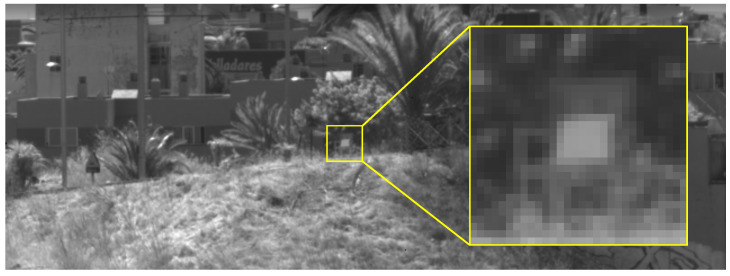
Calibrated reflectance target as seen from DRAGO-2 during the outdoor tests on its 1623 nm band. The target was placed over a chair (highlighted with a yellow square) and tilted until DRAGO-2 gave the desired DN value.

On the 13 October 2023 at noon, DRAGO-2 was placed inside the IACTEC headquarters while pointing outside with no obstructing elements in the light path, not even windows. The day was clear, with no visible clouds, haze or dust in the atmosphere. The calibrated reflectance target mentioned in Section 3.1.1 was then placed 380 m away, oriented so that DRAGO-2 could see it reflecting direct light from the Sun. Then, the target was tilted until the DN value given by DRAGO-2 was in the range of the reference value determined in Section 3.3.4, that is, $y_{r}=4820.1$ DN. In this way, regardless of the actual reflectance value of the target, the light received around 1600 nm would resemble the one received from orbit while pointing to a target with reflectance $\rho_{r}=0.246$. Figure 5 shows the appearance of the target as seen from DRAGO-2, once the tilt adjustment was complete.

With the set-up prepared this way, not only light around 1600 nm would be representative of the conditions seen from orbit but also in the rest of the wavelengths that VINIS-PROTO is sensitive to, if one considers a target with a flat reflectance curve. This is possible because the selected calibrated target has a very flat response as well, as seen in Table 4. In contrast to the conditions that can be reproduced in the laboratory, based on Sun and atmosphere simulators, conducting these tests outdoors ensures the maximum resemblance to the spectrum actually seen in in-orbit conditions. Indeed, if one considers that the atmosphere absorbs light equally in both the downward and the upward directions, the lack of the upward path can be compensated by a greater tilt on the calibrated reflectance target.

**Figure 6:**
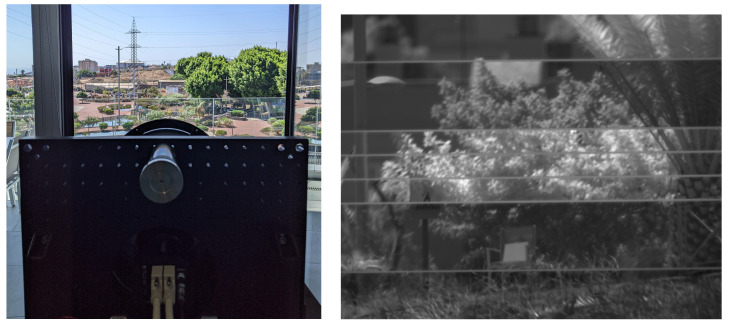
VINIS-PROTO during the outdoor tests. (**a**) Rear view of VINIS-PROTO pointing to the calibrated target from inside the building, next to DRAGO-2 (not seen in the picture). The protective glass that can be seen a few meters away did not obstruct the view to the target, though it may appear so from this perspective. (**b**) Image captured using VINIS-PROTO, with the target falling completely in the SWIR2 band. Note that the location of the filters over the image sensor of VINIS-PROTO were not ordered by wavelength.

Figure 6 shows how VINIS-PROTO was located inside the building pointing to the calibrated target, as well as an example image that it captured during these tests. Note that the horizontal lines in the picture correspond to the separation between the filters over the focal plane. One of the filters is actually divided in three slices, just as mentioned in Table 2.

### 3.5. Data Acquisition

For each of the 5 bands of VINIS-PROTO, bursts of 200 images were acquired and later processed to produce the numbers given in Section 4. The exposure time was set to 700 μs, which is a value where VINIS should not produce a noticeable level of blur while scanning the Earth from LEO. The temperature of the sensor was stabilized to 20 °C using its integrated Thermoelectric Cooler (TEC) in order to avoid the variations in performance that the sensor can have at different temperatures. The frame rate was set to 45 Hz, so each burst of 200 images was acquired in less than 5 s.

As the Sun moved across the sky during the measurements, it was expected that the illumination angle over the target would change, thus changing the amount of received light. This fact was considered during data analysis, but still, the measurements were performed as fast as possible to try to minimize these variations. The total measurement time was 15 min and 34 s.

## 4. Results

This section presents the performance parameters that were derived from the acquired images, more specifically, the SNR and the dynamic range.

### 4.1. Direct Measurements

The first results extracted from the acquired images are listed in Table 10. The only extrapolated values in this table are the simulated reflectances. The term “simulated reflectance” does not refer to the actual reflectance of the calibrated target, but to the $\rho_{t}$ value derived using Equation (4), with an additional correction factor to compensate the slight deviations in reflectance of the calibrated target across the different wavelenghts (see Table 4). In other words, it expresses the reflectance of a target that, when seen from space, would produce the same amount of incoming light as in the present test set-up.

With the help ImageJ 1.54g, an imaging processing software developed by Wayne Rasband (National Institutes of Health, Bethesda, MD, USA) and contributors [45], the set of images corresponding to each band was stacked pixel by pixel to produce per-pixel average maps and per-pixel standard deviation maps. The measured values shown in Table 10 correspond to the spatial average of DSNU-corrected average maps, considering just the region of pixels where the calibrated target fell. The SNR value was obtained by dividing the corrected average maps and the standard deviation maps, and then calculating the spatial average in an analogous way. Finally, the calibration factors in the table were obtained by dividing the measured values and the simulated reflectances. These factors can be used to estimate the output of the sensor for any reflectivity value, as in Equation (4).

### 4.2. Estimated Performance

The objective of this step was to obtain the estimated performance of VINIS-PROTO under the conditions mentioned in Section 3.1. More specifically, the performance parameters that were obtained were the SNR and dynamic range for each band.

Laboratory tests performed with VINIS-PROTO demonstrated that, just like what happened with DRAGO-2, the sensor is highly linear (linearity better than 99%) for practically its whole dynamic range. With the calibration factor in Table 10, it is thus possible to estimate the corrected DN values that would be obtained using VINIS-PROTO for any target. With this information and a proper SNR model, it would be possible to estimate the SNR for any target.

An empirical SNR model was obtained with the VINIS-PROTO sensor by illuminating it in the laboratory at different light levels, covering its whole dynamic range. The SNR value corresponding to each light level was calculated using a similar method as in Section 4.1. Then, a simple polynomial fit was applied to create a model that relates corrected DNs (as measured) and the expected SNR.

Table 11 shows the expected SNR values for the reference reflectance values defined in Section 3.1. In this table, the “worst-case illumination” refers to the maximum SZA expected over Tenerife throughout the year, while the “best-case illumination” refers to the minimum SZA. According to Section 3.1, the later case can make the in-orbit payload receive twice the amount of light with respect to the former. As the test set-up was only prepared to simulate the worst-case illumination; the numbers corresponding to the best-case illumination were obtained by doubling the calibration factors in Table 10, producing an equivalent effect.

In Table 11, the 100% well fill corresponds to the point where the linearity of the sensor deviated over 1%, according to laboratory tests. The well fill is an important number, as it provides an idea of how far the sensor was from saturation under each situation. In investigating the actual detection limits, Table 12 shows the expected dynamic range, understood as the range between the minimum reflectance value that produces the required SNR (SNR >60, as mentioned in Table 1) and the value that produces a 100% well fill under certain illumination conditions.

## 5. Discussion

The final results presented in Section 4.2 represent the pieces of information for which the method described in this paper was conceived. As a reminder, the original intention was to obtain a reliable estimation of the performance that VINIS may have under illumination conditions that are representative of those in orbit. The estimated parameters were the SNR and the dynamic range at each observation band.

### 5.1. Signal-to-Noise Ratio

According to Table 11, VINIS-PROTO managed to reach the required SNR (SNR > 60) under most of the tested circumstances. The only exception is the R (red) band, with an estimated SNR value under the worst-case illumination conditions being just below 60. This result will affect further iterations of the design, which should implement a solution to increase the amount of light in this band. A red filter with higher transmittance than the one used in VINIS-PROTO (see Table 2) should fix this issue.

One unfortunate point during the development of VINIS-PROTO is that it was not possible to procure a proper filter for the B (Blue) band, that is, one that properly rejected any light that does not correspond to blue wavelengths throughout the whole sensitivity range of the sensor. While extended-response InGaAs sensors like the one selected for VINIS are known to be sensitive to wavelengths down to 400 nm, as the sensitivity starts being low near these cut-off wavelengths [20]. Having a proper B filter would have yielded a definite answer as whether VINIS would be able to also properly sense around these shorter wavelengths. The B filter that was finally integrated in VINIS-PROTO does not yield any useful information, as it does not properly reject wavelengths over 1000 nm. While this filter would have been acceptable with silicon-based sensors, which cut around 1000 nm, it is not acceptable in sensors that are sensitive to SWIR wavelenghts.

### 5.2. Dynamic Range

While Table 11 shows promising results in terms of the SNR, Table 12 highlights a possible problem with the dynamic range. Indeed, any observing band will saturate under high reflectance values. This effect is even more remarkable when the illumination conditions are favorable, as the sensor would reach saturation under reflectance values that can easily appear in a typical scene, causing a considerable number of saturated pixels.

For scenes where high reflectance values are expected (e.g., desert), this problem may be solved by reducing the exposure time below its default value, specially when the SZA is expected to be low (e.g., summer season). This imposes more requirements on payload calibration and image post-processing, as these would need to consider variable exposure times. Still, this additional level of complexity would be perfectly manageable.

Besides adjusting the global exposure time, as proposed in the paragraph above, it might be possible to fine-tune the exposure time for each band. Most image sensors, including the one used in VINIS-PROTO, allow defining smaller ROIs than the full frame to perform faster pixel readouts. Exposure times, unfortunately, apply equally to all the pixels, even if they are not read out. Still, one may set a global exposure time and read just a set of bands and then set another global exposure time and read another set of bands. Effectively, this can produce different exposure times for each band.

Finally, it would be worth exploring whether HDR (High Dynamic Range) techniques are applicable to VINIS. This method, which combines images of different exposure times in a single image, may enable VINIS to cover wider dynamic ranges under very variable illumination conditions. The main question here is whether it is possible to read the full frame at a rate that is high enough to capture all ground features in time, considering the fast orbital speeds of LEO.

## 6. Conclusions

This paper described a practical method for obtaining reliable estimations of in-orbit performances of Earth Observation payloads under development. All the steps of the method were explained in the context of an actual space mission, yielding results that have already helped make decisions that will help in subsequent stages of the project. In having relied mainly on actual ground and in-orbit measurements, rather than on complex theoretical models, the risks associated with the innovative parts of this development have been reduced significantly. The key to this method is the possibility of having a reference payload both in orbit and on ground, which is a scenario that is likely to become more and more prevalent, given the expanding accessibility of space and the increasing popularity of small satellites.

Applying the proposed method for the first time has revealed some points of improvement, which could be considered in future test runs. For example, more emphasis can be placed on reducing the length of the test to ensure that the variations in the reflected light during the measurements are kept to a minimum. Ideally, the target should cover all the bands of interest at the same time so there is no need to move the payload to switch between bands. While this may be achieved by bringing the target closer, it needs to be evaluated whether the accuracy of the results would be affected when distances are too short to keep the payload perfectly focused.

## Figures and Tables

**Table 1:** Main specifications of DRAGO-2 and VINIS. Note that SNR is expressed in linear units.

	DRAGO-2	VINIS
Sensor technology	InGaAs	InGaAs (extended range)
Spectral range	1.0 μm to 1.7 μm	0.4 μm to 1.7 μm
GSD @ 500 km	50 m	5 m
Swath @ 500 km	32 km	5 km
Diameter of aperture	33 mm	200 mm
Signal-to-noise ratio (SNR)	>100	>60
Power consumption	5.5 W	35 W
Dimensions	96 mm × 96 mm × 170 mm	650 mm × 400 mm × 400 mm
Weight	1.26 kg	<20 kg

**Table 2:** Properties of the filters used in each band of VINIS-PROTO, ordered by central wavelength. The transmittance in the pass bands is expressed in linear units (%), while the transmittance in the rejection bands is expressed in base-10 logarithmic units (OD, “Optical Density”).

Band Name	Central λ	Bandwidth	Transmittance	Rejection	Notes
B (Blue)	490 nm	75 nm	≈98%	OD > 5	Leaks for λ>1000 nm
R (Red)	640 nm	100 nm	≈90%	OD > 4	
NIR	850 nm	100 nm	≈85%	OD > 4	
SWIR1	1060 nm	100 nm	≈65%	OD > 5	Divided in 3 smaller slices
SWIR2	1640 nm	200 nm	>90%	OD > 5	Response cut by the sensor itself

**Table 3:** Comparison of the two observing bands used for the reference images.

	DRAGO-2 @ ION SCV-007	Sentinel-2 B11
Central λ	1623 nm	1610 nm
Bandwidth	135 nm	90 nm
Resolution	65 m (@ 650 km)	20 m

**Table 4:** Reflectance values of the calibrated ground target for the bands of VINIS-PROTO, ordered by central wavelength.

Band Name	Central λ	Reflectance
B (Blue) ^1^	490 nm	18.3%
R (Red)	640 nm	19.3%
NIR	850 nm	20.8%
SWIR1	1060 nm	21.8%
SWIR2	1640 nm	24.1%

^1^ Band “B” of VINIS-PROTO leaks over 1000 nm.

**Table 5:** Sensibility analysis of the radiance received by an in-orbit payload for a relevant set of related parameters, as derived from the references specified in the table. The irradiance loss was calculated as the relative difference between the reference and test values. Only one parameter was changed at a time. The selected reference and test values represent the ranges that could be expected while observing locations at mid-latitudes, thus covering the case under study. “AU” stands for “Astronomical Units”.

Parameter	Reference Value	Test Value	Radiance Loss	Reference
Target reflectance	1	0	100%	[29]
Illumination angle	0°	60°	59.6%	[32]
Target altitude	3 km	0 km (sea level)	28.3%	[33]
Visibility (due to aerosols)	50 km (very clear)	2 km (very hazy)	17.9 %	[29]
Earth–Sun distance	0.9833 AU (perigee)	1.0167 AU (apogee)	6.5%	[31]
Atmospheric model	Mid-latitude winter	Mid-latitude summer	4.5%	[29]

**Table 6:** Comparison of the two observing bands used for the reference images.

	DRAGO-2	Sentinel-2A
Distance between ROI and orbit footprint	30 km	15.5 km
Orbit altitude during pass	653 km	793 km
Observation Zenith Angle (OZA) (nadir: 0°)	2.63°	1.12°
Atmospheric attenuation factor	0.9997	0.9999

**Table 7:** DEM-related statistics along the ROI.

	Min.	Avg.	Max.	Std.
Elevation [m]	1279.3	1287.4	1296.3	4.545
Slope [°]	0.000	1.771	3.835	0.924

**Table 8:** Sun coordinates as seen from the ROI at the time of each pass.

	DRAGO-2	Sentinel-2A
Azimuth	136.24°	153.67°
Elevation	35.16°	42.41°
SZA (90° − elevation)	54.85°	47.59°
Attenuation factor (cos(SZA))	0.576	0.674

**Table 9:** Imaging-related statistics along the ROI, all around 1600 nm wavelength.

	Min.	Avg.	Max.	Std.
DRAGO-2 [corrected DN ^1^]	4768.7	4820.1	4870.1	27.816
Sentinel-2A L2A B11 (SR reflectance)	0.238	0.246	0.253	0.003

^1^ Corrected from DSNU (Dark Signal Non-Uniformity) and PRNU (Photon Response Non-Uniformity).

**Table 10:** Direct measurements obtained with VINIS-PROTO in outdoor tests. The term “simulated reflectance” refers to the reflectance of a target that, when seen from space, would produce the same amount of incoming light as in the present test set-up. On the other hand, the term “measured value” refers to direct measurements obtained using the sensor of VINIS-PROTO, with native output units that are 14-bit digital numbers (DNs).

Band Name	Central λ	Simulated Reflectance	Measured Value [DN]	SNR	Calibration Factor [DN]
B (Blue) ^1^	490 nm	0.184	9103.78	191.153	49,413.187
R (Red)	640 nm	0.198	2907.83	95.939	14,697.897
NIR	850 nm	0.207	3993.31	120.496	19,247.145
SWIR1	1060 nm	0.221	3814.90	116.003	17,285.115
SWIR2	1640 nm	0.250	3570.19	109.927	14,281.186

^1^ Band “B” of VINIS-PROTO leaks for λ>1000 nm.

**Table 11:** Estimated SNRs of VINIS-PROTO while pointing to targets with typical reflectance values, covering two corner cases of illumination geometries. A 100% well fill indicates saturation of the image sensor.

			Worst-Case Illumination		Best-Case Illumination	
Band Name	Central λ	Reflectance	SNR	Well Fill	SNR	Well Fill
B (Blue) ^1^	490 nm	0.10	135.08	41.88%	206.78	83.75%
R (Red)	640 nm	0.10	59.63	12.46%	97.30	24.91%
NIR	850 nm	0.25	132.87	40.78%	203.38	81.56%
SWIR1	1060 nm	0.25	124.27	36.62%	190.44	73.24%
SWIR2	1640 nm	0.25	110.24	30.26%	169.56	60.51%

^1^ Band “B” of VINIS-PROTO leaks for λ>1000 nm.

**Table 12:** Estimated dynamic range of VINIS-PROTO for the selected sensor configuration, covering two corner cases of illumination geometries.

		Worst-Case Illumination		Best-Case Illumination	
Band Name	Central λ	$\rho_{\min}$	$\rho_{\max}$	$\rho_{\min}$	$\rho_{\max}$
B (Blue) ^1^	490 nm	0.030	0.239	0.015	0.119
R (Red)	640 nm	0.101	0.803	0.050	0.401
NIR	850 nm	0.077	0.613	0.038	0.307
SWIR1	1060 nm	0.086	0.683	0.043	0.341
SWIR2	1640 nm	0.104	0.826	0.052	0.413

^1^ Band “B” of VINIS-PROTO leaks for λ>1000 nm.

## Data Availability

The third-party satellite imagery used throughout the document comes from Copernicus, the European Union’s Earth Observation programme [39,41,43]. The reference DRAGO-2 image was obtained by the IACTEC-Space group and then published as part of its first set of in-orbit imagery (https://www.iac.es/en/outreach/news/drago-2-makes-its-first-observations-space (accessed on 16 November 2023)). The processed image, just as used as part of the present study, is provided in the Supplementary Material.

## Supplementary Materials

The following supporting information can be downloaded at: https://www.mdpi.com/article/10.3390/s24103160/s1, Figure S1: Reference DRAGO-2 image, just as used as part of the present study. The image is in 32-bit per pixel GEOTiff format. It was georeferenced using the QGIS software (version 3.28.15), based on a projective transform with Lanzcos resampling. The image includes DSNU (Dark Signal Non-Uniformity) and PRNU (Photo Response Non-Uniformity) corrections, but no bad pixel corrections. Some image visualization programs may have issues opening this file. We recommend to open the file with QGIS.

## Abbreviations

The following abbreviations are used in this manuscript:

ADC	Analogue-to-Digital Converter
AM	Air Mass
AU	Astronomical Unit
CCD	Charge-Coupled Device
CDTI	Centro para el Desarrollo Tecnológico y la Innovación
DEM	Digital Elevation Map
DN	Digital Number
DRAGO	Demonstrator for Remote Analysis of Ground Observations
DSNU	Dark Signal Non-Uniformity
DUT	Device Under Test
EO	Earth Observation
ERDF	European Regional Development Fund
ESA	European Space Agency
GIS	Geographic Information System
GSD	Ground Sampling Distance
HDR	High Dynamic Range
IAC	Instituto de Astrofísica de Canarias
IACTEC	IAC Tecnología
IR	Infrared
MEDI	Marco Estratégico de Desarrollo Insular
MSI	[Sentinel-2] MultiSpectral Instrument
NIR	Near-Infrared
OD	Optical Density
OZA	Observation Zenith Angle
PRNU	Photo Response Non-Uniformity
ROI	Region Of Interest
SNR	Signal-to-Noise Ratio
SR	Surface Reflectance
SWIR	Short-Wavelength Infrared
SZA	Solar Zenith Angle
TEC	Thermoelectric Cooler
TLE	Two-Element Set
TOA	Top-Of-Atmosphere
VINIS	Visible Near-Infrared and SWIR
