# Supplementary figures and images for: Performance Estimation of a Medium-Resolution Earth Observation Sensor Using Nanosatellite Replica
Source: Sensors (Basel). 2024 May 16;24(10):3160. doi: 10.3390/s24103160 (PMC11125122; doi:10.3390/s24103160)

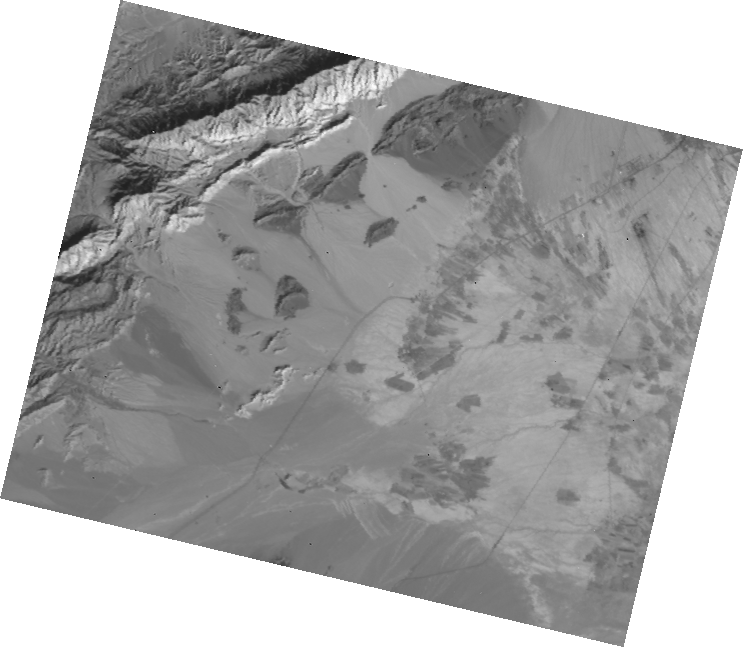

Supplement: Supplementary file 1 [file sensors-24-03160-s001.zip › Figure S1 drago2_ref_geo_preview.png]
